# Is Breath Best? A Systematic Review on the Accuracy and Utility of Nanotechnology Based Breath Analysis of Ketones in Type 1 Diabetes

**DOI:** 10.3390/bios15010062

**Published:** 2025-01-19

**Authors:** Kamal Marfatia, Jing Ni, Veronica Preda, Noushin Nasiri

**Affiliations:** 1 Faculty of Medicine, Health and Health Sciences, Macquarie University, Level 3, 75 Talevera Road, Macquarie Park, NSW 2113, Australia; jing.ni@mqhealth.org.au (J.N.); veronica.preda@mqhealth.org.au (V.P.); 2 NanoTech Laboratory, School of Engineering, Faculty of Science and Engineering, Macquarie University, Sydney, NSW 2109, Australia; noushin.nasiri@mq.edu.au; 3 Smart Green Cities Research Centre, Macquarie University, Sydney, NSW 2109, Australia

**Keywords:** nanotechnology, breath analysis, acetone, ketones, DKA, type 1 diabetes

## Abstract

Timely ketone detection in patients with type 1 diabetes mellitus (T1DM) is critical for the effective management of diabetic ketoacidosis (DKA). This systematic review evaluates the current literature on breath-based analysis for ketone detection in T1DM, highlighting nanotechnology as a potential for a non-invasive alternative to blood-based ketone measurements. A comprehensive search across 5 databases identified 11 studies meeting inclusion criteria, showcasing various breath analysis techniques, such as semiconducting gas sensors, colorimetry, and nanoparticle-based chemo-resistive sensors. These studies report high sensitivity and correlation between breath acetone (BrAce) levels and blood ketones, with some demonstrating accuracies up to 94.7% and correlations reaching R^2^ values as high as 0.98. However, significant heterogeneity in methodologies and cut-off values limits device comparability and precludes meta-analysis. Despite these challenges, the findings indicate that BrAce monitoring could offer significant clinical benefits by enabling the earlier detection of ketone buildup, reducing DKA-related hospitalisations and healthcare costs. Standardising BrAce measurement techniques and sensitivity thresholds is essential to broaden clinical adoption. This review underscores the promise of nanotechnology-based breath analysis as a transformative tool for DKA management, with potential utility across varied ketotic conditions.

## 1. Introduction

Diabetes is a significant public health challenge, affecting approximately 1 in 20 Australians and ranking as the seventh leading cause of mortality in the country in 2020 [1,2]. Inadequately managed diabetes presents severe risks, both acute and long-term, contributing to a spectrum of microvascular and macrovascular complications [3]. These complications impose a substantial financial burden on the Australian healthcare system, with associated costs estimated at $3 billion in 2019 [4]. Addressing the clinical and economic impact of diabetes necessitates innovative approaches to diagnosis and management, highlighting the importance of advancing technologies for early detection and effective monitoring.

For individuals with T1DM, insufficient levels of insulin hinder the utilisation of glucose for energy, causing the body to break down fat as an alternative energy source [5]. Reduced glucose availability, either from fasting, a ketogenic diet, insulin deficiency, or insulin resistance, prompts the body to produce increased amounts of acetyl CoA from fatty acids [5,6]. This process results in elevated concentrations of ketone bodies in both blood and breath. Diabetic ketoacidosis (DKA) occurs when ketone bodies accumulate rapidly due to impaired insulin action and reduced glucose utilisation [5,6]. This condition often necessitates acute care, leading to emergency department (ED) and intensive care unit (ICU) admissions, and is associated with substantial morbidity, potential mortality, and significant costs to the healthcare system. Addressing these clinical challenges underscores the need for efficient and accessible diagnostic tools for the early detection and management of DKA [2]. According to data from the International Diabetes Federation, in 2013, over 79,000 children were diagnosed with type 1 diabetes (T1D), with a staggering 80% presenting with DKA at the time of diagnosis [7]. Moreover, studies highlight that social determinants of health are strong predictors of DKA recurrence [8].

Breath analysis has emerged as a non-invasive alternative for managing diabetes mellitus and DKA, focusing on detecting volatile organic compounds (VOCs), particularly acetone, as biomarkers [9,10,11]. Acetone concentration in exhaled breath shows promise as a novel biomarker for non-invasive diabetes diagnostics and monitoring, particularly for type I diabetes [12,13]. The elimination of free acetone from the lungs follows the principles of diffusion, with acetone levels in exhaled air being approximately 1/330 of the acetone concentration in plasma [14]. In healthy individuals, breath acetone (BrAce) levels typically range from approximately 0.3 to 1.0 parts per million (ppm) [10,11]. In contrast, individuals with T1DM may exhibit higher BrAce levels, especially when ketosis is present, typically >1.7 ppm [15]. The specific levels can vary depending on factors such as the individual’s metabolic state, dietary habits, and overall health [14,15].

Early studies focused on gas chromatography–mass spectrometry (GC-MS) for acetone detection. GC-MS offers high accuracy and specificity but is limited by its cost, time consumption, and lack of portability, restricting its clinical utility [10,11]. Advances in sensing technologies, including nanotechnology-based devices, now enable faster, cost-effective, and portable breath analysis solutions, addressing the limitations of earlier methods [11,16,17,18]. In the context of diabetes management, sensors operate through mechanisms such as chemo-resistive [10,11], colorimetric [19,20], and optical detection [21]. Among them, highly sensitive chemo-resistive-based sensors, such as SnO_2_, MoO_3_, WO_3_, and NiO, have attracted significant attention as they exhibit the capability to detect a wide range of gases with remarkable sensitivity [9,10,11,22,23]. The fundamental working principle of metal oxide semiconductor-based gas sensors, whether p-type or n-type, revolves around surface interactions with the target analyte [10,11]. Initially, surface oxygen species create a uniform electron depletion layer, resulting in high resistance. However, exposure to gases containing target analytes triggers surface reactions that neutralise these oxygen species, thereby lowering the resistance, facilitating detection [10,11].

Recent developments in nanotechnology have led to the creation of highly sensitive and selective sensors for breath acetone detection. Jiang et al. [24] developed a highly sensitive mixed potential-type acetone sensor for breath analysis, targeting diabetic ketosis diagnostics. The sensor uses a Gd_2_Zr_2_O_7_ solid electrolyte combined with a CoSb_2_O_6_ sensing electrode, fabricated through a sol–gel method. This novel configuration achieves an ultralow detection limit of 10 ppb and provides linear detection across a wide concentration range of 10 ppb to 100 ppm, with excellent selectivity, repeatability, and stability, even under varying humidity levels. The sensor’s performance was validated using breath samples from healthy individuals and diabetic patients, demonstrating a strong correlation between sensor response and acetone concentration measured by gas chromatography–mass spectrometry. Additionally, the sensor showed high accuracy in distinguishing diabetic ketosis patients, making it a promising tool for non-invasive diabetes management and blood ketone monitoring.

Li et al. [25] developed a wearable, wireless facemask platform for real-time acetone detection in breath, aimed at monitoring lipid metabolism non-invasively. The facemask integrates a Ti_3_C_2_T_x_ MXene-based sensor functionalized with in situ-grown TiO_2_ nanoparticles and short peptides, enhancing sensitivity, selectivity, and response calibration through light irradiation. The platform incorporates a textile filter with Pt nanoparticles for breath interference filtration, achieving an acetone detection limit of 0.31 ppm. On-body tests validated the sensor’s ability to monitor dynamic changes in breath acetone during exercise and dietary interventions, demonstrating its potential for personalised healthcare in lipid metabolic management. This innovative system represents a promising step toward integrating MXene-based sensors into daily-used textiles for real-time, non-invasive health monitoring.

In another approach [26], a flexible pre-concentrator device was developed for acetone detection in human breath, using modified metal–organic framework (MOF) materials [23] embedded in a wearable face mask. By coating MIL-101 (Cr) nanoparticles with polydimethylsiloxane (PDMS) through physical vapour deposition, an enhanced hydrophobicity and gas adsorption capacity was achieved, enabling efficient acetone pre-concentration under high-humidity conditions [26]. The device demonstrated a detection range from 100 ppb to 2500 ppb, with a 76.3-fold signal enhancement compared to commercial materials. Integrated with a mass spectrometer, this system achieved linear and sensitive acetone quantification, showcasing potential for non-invasive health monitoring applications in flexible electronic systems. While current nanostructured gas sensors generally operate at sub-ppm detection limits [10,11,17,18], they fall short of the ppb-level sensitivity required for effective healthcare applications. However, recent developments in sensors incorporating noble or minor metal modifications offer a promising pathway toward practical, user-friendly diagnostic devices that could be integrated into patient homes, clinics, and hospitals. These advancements underscore the potential of nanotechnology in developing non-invasive, efficient, and accessible diagnostic tools for the early detection and management of diabetes and its complications, like diabetic ketosis. By enhancing the sensitivity, selectivity, and portability of breath analysis devices, nanotechnology-based sensors represent a promising frontier in diabetes care. Breath analysis has the potential to facilitate timely interventions and improved patient outcomes, making breath the best future option for non-invasive monitoring in diabetes.

## 2. Methods

### 2.1. Protocol

This systematic review was conducted according to the Preferred Reporting Items for Systematic Reviews and Meta-Analyses (PRISMA) framework and Cochrane Handbook guidelines [27]. The protocol for the study was registered with the PROSPERO International Register of Systematic Reviews (Registration ID: CRD42023410996).

### 2.2. Search Strategy

A comprehensive search was conducted across 5 databases—Medline, SCOPUS, Embase, Cochrane, and PubMed—to evaluate the efficacy and utility of nanotechnology-based breath analysis devices in the T1DM population. Additional references were examined to ensure the inclusion of all relevant evidence. The search strategy utilised medical subject headings (MeSHs) such as “diabetic ketoacidosis” and incorporated Boolean operators to refine and merge search terms (details in Appendix A). A systematic approach was employed, framing the research question using the PICO framework: the population comprised T1DM patients, both with and without DKA; the intervention focused on BrAce measurement; the comparators included blood capillary ketone testing, or TKB; and the outcomes assessed the accuracy of BrAce measurements, their correlation with blood ketone levels, and their predictive value in the onset and resolution of DKA. This structured methodology ensured a rigorous and targeted review of the existing literature.

A diverse range of keyword combinations was utilised across the 5 databases to maximise the retrieval of relevant studies. This flexible approach, rather than relying on a standardised keyword set, was designed to capture a broad spectrum of articles, ensuring comprehensive coverage of both nanotechnology-based breath analysis techniques and other breath analysis technologies. Such a strategy aligns with the review’s objective of critically comparing the applications of nanotechnology in ketone detection to existing breath analysis methods, thereby providing a holistic perspective on the field.

### 2.3. Study Selection and Eligibility Criteria

The inclusion criteria encompassed studies published within the past 10 years, in English, that measured BrAce, and focused specifically on the T1DM population. TKB measured in blood samples served as the gold standard for comparison across all included studies. Exclusion criteria eliminated studies conducted on animal models, those not published in English, studies that did not include breath samples from T1DM patients, and those focusing solely on T2DM populations. This screening process initially identified 291 articles, encompassing cohort, cross-sectional, and prospective study designs. After removing 86 duplicates, 205 unique studies remained for further analysis.

### 2.4. Quality Assurance

Two researchers, K.M. and J.N., independently conducted database searches using predefined criteria and agreed-upon search terms. The Rayyan platform was employed to efficiently identify and remove duplicate records. The consolidated search results were subsequently reviewed and assessed by K.M., J.N., and V.P. This evaluation included a rigorous appraisal of research quality, informed by prior reviews, the researchers’ collective expertise, and the National Heart, Lung, and Blood Institute (NHLBI) Quality Assessment Tool. Any discrepancies were resolved through group discussions with the research supervisor, V.P. The screening process involved a sequential review of titles and abstracts followed by full-text evaluation, with exclusion decisions supported by clearly documented justifications.

### 2.5. Data Extraction

The primary reviewer, K.M., independently conducted data extraction using a customised Excel tabulation form based on the Cochrane Consumers and Communication Review Group’s data extraction template. This systematic approach enabled the detailed documentation of key study characteristics, including objectives, methods, participant demographics, technology used, study optimisation processes, primary and secondary outcomes, study design, limitations, BrAce detectability limits, and comparators. The second reviewer, J.N., and the supervisor, V.P., subsequently cross-referenced and validated the extracted data to ensure accuracy and consistency.

### 2.6. Risk of Bias Tools

The risk of bias and quality assessment was conducted by the primary reviewer using an Excel spreadsheet and the National Heart, Lung, and Blood Institute (NHLBI) Quality Assessment Tool for Observational Cohort and Cross-Sectional Studies as seen in Appendix C. This tool facilitated the evaluation of internal validity and potential bias in the selected studies by examining critical aspects such as study design, confounding variables, and outcome measurement methods, ensuring the inclusion of high-quality evidence in the final review. The tool comprised 14 questions, each rated as “yes”, “no”, or “not applicable (N/A)”, to assess internal validity. Studies were categorised as poor (≤5/14), fair (≥6/14), or good (≥10/14) based on their scores. While no studies were excluded solely based on quality, these ratings were taken into account during data analysis and the interpretation of findings. The included studies demonstrated a range of quality from poor to good, with an overall low risk of bias for cohort and cross-sectional studies, as detailed in Appendix B.

The risk of bias assessment allowed us to incorporate study quality into data interpretation. While two studies were rated as “poor”, their findings were contextualised within the broader framework of higher-quality evidence to maintain balanced conclusions. The majority of studies (four rated as “good” and five as “fair”) demonstrated a low to moderate risk of bias, supporting the reliability of our overall conclusions. Collectively, the evidence suggests that nanotechnology-based breath analysis holds significant clinical potential for ketone detection in T1DM. However, it also highlights the need for more rigorous research in this emerging field. The observed variability in study quality necessitates caution when interpreting results, particularly from studies with “poor” quality ratings. This variability informed our decision to forego a meta-analysis and instead adopt a qualitative synthesis approach. The risk of bias assessment underscores the urgent need for high-quality, large-scale studies to solidify the evidence base for breath analysis as a non-invasive tool for monitoring DKA.

### 2.7. Synthesis of Results

**Figure 1:**
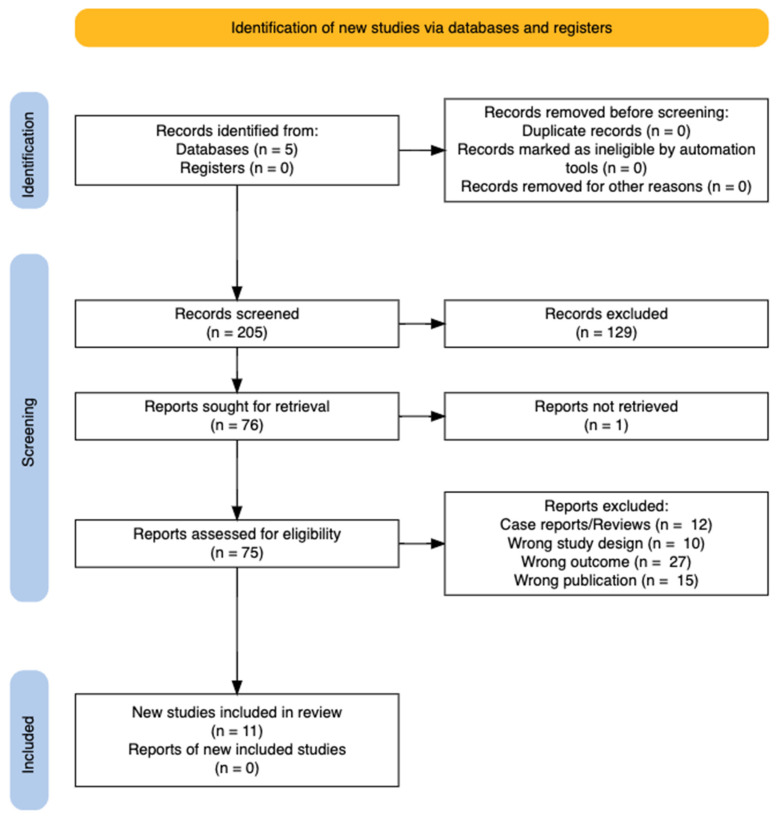
PRISMA flow chart showing final results.

The study selection process is detailed and visually represented through a PRISMA 2020 flowchart (Figure 1), illustrating each step in the screening and inclusion of articles. Beginning with the identification of numerous studies, the application of defined inclusion and exclusion criteria refined the selection to focus on studies relevant to the research objectives. The primary outcome of interest was the accuracy of assessing the risk of DKA development in patients with T1DM based on BrAce levels and their correlation with TKB or beta-hydroxybutyrate (BHB) levels. Across the included studies, linear regression analyses consistently demonstrated a positive correlation between BrAce and blood ketone levels. However, some heterogeneity was observed, likely stemming from variations in the devices used, sample sizes, and study populations. These factors highlight the potential influence of methodological differences on the strength of the observed correlations and underscore the need for standardised approaches in future research.

## 3. Results

### 3.1. Search Yield

A total of 291 studies were identified through the primary database search, with no additional studies found via snowballing or reference list reviews. After removing duplicates, 205 unique articles remained, of which 129 were excluded following title and abstract screening. One article was deemed irretrievable. The remaining 75 articles underwent full-text screening, resulting in 11 studies being included in the final analysis. Exclusions were based on publication type, such as reviews and case reports (12); inappropriate study design (10); irrelevant outcomes measured (27); and wrong population (15). The final 11 studies underwent critical appraisal and comprised three cohort studies, three cross-sectional studies, one exploratory study, one prototype development study, one proof-of-concept study, one methodological study, and one comparative study.

### 3.2. Demographic and Clinical Characteristics of Included Studies (Table 1)

The included studies can be broadly categorised into three groups: newly developed nanosensing technologies, evaluations of existing technology accuracy, and comparisons between laboratory-based and portable devices. The review found that most studies measuring BrAce in individuals with T1DM were conducted on relatively small cohorts, with the exception of two larger studies (Hancock 2020 and Blaikie 2014) [28,29]. Across all studies, a total of 374 participants were included, with reported BrAce levels ranging from 0.02 to 474 ppm. The review incorporated studies employing various techniques, with a subset utilising nanotechnology-based methods such as nanoparticle-based chemo-resistive sensors and semiconducting gas sensors, which exploit nanoscale materials for superior gas sensing performance. Other methods, including ringdown spectroscopy, colorimetry, and gas chromatography, were also included due to their reported sensitivity and applicability in detecting BrAce in T1DM patients, though they were not classified as nanotechnology based. The studies were conducted across diverse geographic locations, including Malaysia, the USA, Japan, the UK, the Netherlands, Switzerland, and Taiwan.

### 3.3. Correlation Between TKB and BrAce

In the largest study, which included 113 participants, breath acetone (BrAce) demonstrated a stronger correlation with TKB than with blood glucose levels (R^2^ = 0.29 versus R^2^ = 0.039, respectively) in detecting DKA [29]. Across all included studies, a robust association was observed between BrAce concentrations and blood ketone levels, particularly β-hydroxybutyrate (BHB). For example, Akturk (2021) reported statistically significant results with a *p*-value of 0.0066, and Tsunemi (2022) documented a strong correlation with an R-value of 0.828 [30,31]. This consistent correlation among individuals with T1DM highlights the diagnostic potential of BrAce measurements. Furthermore, all breath analysis devices evaluated demonstrated the ability to distinguish between healthy individuals and those with T1DM. However, Güntner (2022) noted that their sensors underpredicted acetone levels at high BrAce concentrations due to the non-linear diffusion of the analyte within sensor films and its adsorption on nanoparticle surfaces [32]. This underlines the importance of enhancing the accuracy of breath analysis systems through improved sensor calibration algorithms. These findings emphasise that refining the technology is essential to realise its full clinical applicability [29].

**Table 1:** Characteristics of included studies and finding.

Study		Population		Exposure			Outcomes				
Ref.	Country	Sample Size (n)	Mean Age or Range (Years)	T1DM	Technology/Device	Comparative Measure	Can Differentiate Breath of Ketosis and Non-Ketosis State	Detection Range	Sensitivity and Specificity	BrAce and TKB (R^2^)	Conclusions
[33]	Malaysia	3		Y	Semiconductor FIGARO TGS 822	Blood ketone levels	Y	3–7 PPM		0.92	- There is a good correlation between BrAce and TKB in blood.
[14]	USA	32		Y	Ringdown spectroscopy	Blood ketone levels	Y	0.13–3.97 PPM			- The device can distinguish between the breath of diabetic and healthy patients. - Based on the background subtraction method, acetone is the only significant gas measurable at 266 wavelengths. - Alcohol consumption may generate a false positive signal for BrAce.
[13]	Malaysia	35	10–80	Y	Colorimetry	Blood ketone levels and portable ketoscan device	Y	0.02–50 PPM		0.98	- The highly selective and sensitive colorimetric sensor has a smartphone-assisted unit to analyse the breath of human subjects, and can predict the concentration of acetone. - The proposed device showed more accuracy compared to the commercial Ketoscan device.
[31]	Japan	35	40–57	Y	Semiconductor gas sensor-FM-001	Blood ketone levels and another semiconducting gas sensor	Y	538–15000 PPB	Sens = 73.3%, Spec = 100%	0.69	- BrAce strongly correlates with TKB (correlation was stronger in patients whose serum C-peptide was not low). - BrAce is good for detecting DKA but not good for detecting severe DKA or for those that drink alcohol. - When BrAce > 3400 ppb, there is a high risk of proceeding to DKA.
[28]	UK	81		Y	Ion Molecule Reaction Mass Spectrometry	Blood ketone levels using Abbott Freestyle Optium Meter	Y	0.25–474 PPM	Sens = 91%	0.85	- BrAce falls more gradually than TKB during the resolution of ketosis. - Patients were divided into three risk categories (normal, elevated and high) of developing DKA based on their BrAce. - The elimination of acetone in breath is the slowest (rate-determining) step, involving the kinetics of formation and loss of acetone.
[34]	USA	21	43	Y	metal oxide semiconductor sensors PBAM	Mass spectroscopy and blood ketone levels	Y	0–45 PPM	Sens = 83% Spec = 80%	0.97	- The relatively high daily variability of ketone levels indicate that single blood or BrAce measurements are often not sufficient to assess daily ketone exposure for most users. - Single coincident blood and BrAce measurements show only a moderate correlation, possibly due to the temporal lag between BrAce and blood BHB. - Vigorous or prolonged exercise can cause an increase in ketone levels in the hours following exercise.
[35]	Taiwan	12		Y	Transform-GC-MS	Blood ketone testing and urine	Y	0.1–100 PPM			- The concentration of acetone in breath from a healthy subject is extremely low compared to a diabetic patient.
[7]	Netherlands	4		Y	Quantum cascade laser-based spectroscopic system	Blood ketone testing- Medi Sense Precision Xceed	Y	0.05- 3 PPM			- The acetone concentration of minors with T1D is lower than those measured in adults. - Where the ketone levels remain low, the acetone levels in breath do not change considerably; this is due to the lag in acetone diffusion from plasma to the lungs.
[32]	Switzerland	19	20–36	Y	chemo resistive sensor and nanoparticles	Blood ketone testing and mass spectrometer (PTR-TOF-M)	Y	271–3364 PPB		0.9	- The sensor tends to underpredict BrAce, but only at high normalised BrAce. - The sensor can track BrAce dynamics during fasting, exercising, and OGTT. - Large inter-subject variation has been observed, which reflects differences in the activation of fatty acid oxidation or cardiorespiratory fitness.
[30]	USA	19	24.1 ± 12.2	Y	colorimetry	Blood ketone testing	Y	1–60 PPM	Sens = 94.7% Spec = 54.2%		- The BrAce measurements were significantly associated with elevated TKB in adults, but not in fasting adults or in children.
[29]	UK	113	7–18	Y	soft-ionization mass spectrometer	Abbott ketone blood testing	Y	0–3 PPM		0.29	- BrAce levels were found to increase with TKB levels, and a significant relationship was found between the two. - Single BrAce measurements do not provide a good measure of BGLs. - BrAce concentrations show wide variations amongst healthy individuals and depend subtly upon diet and time of day.

## 4. Discussion

In investigating the accuracy and utility of nanotechnology-based breath analysis for ketone detection in T1DM, several key findings have emerged, highlighting the complexities involved in interpreting BrAce levels.

### 4.1. The Time Lag Effect Between TKB and BrAce

A shared finding across studies by Hancock (2020), Güntner (2022) and Suntrup (2020) is that acetone elimination from breath is a slow process, often resulting in detectable acetone levels even after ketosis has resolved [28,32,34]. This emphasises the importance of understanding the kinetics of acetone elimination for the accurate interpretation of BrAce measurements. Furthermore, Hancock (2020) observed that changes in BrAce levels become significant only at relatively high blood ketone concentrations. This aligns with the understanding that the diffusion of acetone from plasma to the lungs is strongly influenced by the concentration of acetone in the blood [14,28]. These insights highlight the nuanced relationship between BrAce levels and blood ketones, emphasising the need for careful consideration in clinical applications. This highlights the critical need for improved calibration methods or algorithms to address the time lag between blood ketone levels and BrAce measurements. Moreover, several studies reported a lack of data on higher blood ketone concentrations, particularly in individuals with DKA. An exception is Hancock (2020), which included subjects with βHB levels ranging from 0.1 to 7.6 mmol/L and BrAce levels from 0.3 to 474 ppm [28]. This limitation may hinder the comprehensive understanding of acetone measurements in critical medical scenarios involving elevated TKB levels, underscoring the importance of further research in this area. In practical terms, BrAce nanotechnology proves highly effective for detecting the onset of DKA, offering an early warning system for healthcare providers and patients [7,28,34]. However, for monitoring the recovery and resolution of DKA, blood ketone measurements remain the more accurate and responsive method [15]. While BrAce technology provides valuable insights, it is best utilised in combination with other monitoring methods to ensure a comprehensive assessment of DKA progression and the effectiveness of treatment.

### 4.2. Addressing Factors That Affect BrAce

Many of the studies shared common limitations, including potential false positive signals in breath analysis resulting from alcohol consumption, fasting, exercise, time of sample collection, and tooth brushing [14,29,30,31,32,34]. Furthermore, the findings highlight the need to move beyond simply measuring acetone concentration. To develop a comprehensive, adaptable, and personalised point-of-care breath analysis system, researchers must account for intrasubject variance factors such as diet, alcohol intake, insulin usage, glycogen reserves, and overall fitness when designing the system’s algorithm. The limited sample sizes of previous studies have posed challenges in establishing and validating a breath analysis framework that accommodates the numerous variables influencing exhaled acetone levels. For instance, a study by Güntner (2022) identified significant inter-subject variation in BrAce levels, potentially driven by differences in glycogen stores, cardiorespiratory fitness, and metabolic fuel preferences [32]. These findings underscore the importance of incorporating a multifactorial approach in future system designs. All studies collectively underscore the critical need for further research and validation to fully establish the clinical utility and real-world applicability of BrAce measurements. This includes studies with larger sample sizes, extensive longitudinal investigations at higher ketone ranges, and personalised research focusing on paediatric populations, who are at the highest risk for developing DKA [36]. Developing a breath analysis system with high selectivity necessitates a sensor matrix that is highly specific to acetone while repelling other VOCs and gases. Such a system must accurately measure BrAce concentrations under varying conditions of humidity, pressure, temperature, and the presence of interfering analytes [37].

In the study by Tsunemi et al. (2022) [31], participants were instructed to gargle prior to measurement to mitigate interference from isoprene, a naturally occurring compound in the oral cavity. Similarly, another study advised participants to avoid tooth brushing for at least two hours and alcohol consumption for 24 h before testing to minimise exogenous influences [32]. These precautions highlight the inherent challenges of measuring breath acetone solely from exhaled air due to contaminants and gases in the ambient environment [37]. Achieving accurate detection amidst these interferences requires advanced sensor selectivity and sensitivity, making the task inherently complex. A promising solution could involve integrating artificial intelligence to personalise the correlation between sensor responses and a patient’s unique metabolic or disease state. This approach has the potential to enable the development of a highly accurate, personalised point-of-care BrAce system.

### 4.3. Single Standalone Measurements of BrAce Are Not Sufficient to Assess Ketone Levels

The current literature highlights the dynamic and variable nature of BrAce levels in daily life [29,34]. Blaikie et al. [29] reported significant fluctuations in BrAce concentrations among healthy individuals, attributing this variability to factors such as diet and time of day. Similarly, Suntrup et al. [34] emphasised the considerable daily variability in ketone levels, noting that isolated measurements of blood or BrAce often fail to effectively capture an individual’s overall daily ketone exposure. These variations underscore the limitations of single measurements, which may not reliably reflect an individual’s ketone profile. Suntrup et al. further suggested that, for most users, single-point measurements of blood or BrAce are insufficient to provide a comprehensive assessment of daily ketone exposure, calling for more integrated or frequent monitoring approaches [34]. Dietary choices, insulin regimens, and individual responses to lifestyle factors introduce significant complexities, underscoring the need for a more tailored and individualised approach to BrAce measurement [29,33,34]. The limitations of single measurements extend beyond technical constraints, reflecting the intricate interplay of physiological and environmental factors that influence BrAce dynamics [38]. Collectively, these studies highlight the inadequacy of standalone measurements in capturing the nuanced and dynamic nature of a patient’s ketone levels, advocating for a more holistic and personalised approach to BrAce assessment.

### 4.4. Euglycemic DKA Risk from SGLT-2 Inhibitor Use

The widespread adoption of SGLT2 inhibitors has revolutionised diabetes management, providing significant benefits in blood sugar control and reducing cardiovascular risks. However, this class of medication is associated with a two-fold increased risk of euglycemic DKA, a condition where ketosis occurs despite normal blood sugar levels [39,40]. This presents a diagnostic challenge, as conventional blood and urine tests often fail to detect early signs of ketosis, increasing the risk of patients unknowingly progressing toward DKA. Breath acetone analysis offers a promising solution by providing immediate insights into the metabolic state of diabetic patients, including those on SGLT2 inhibitors, enabling earlier detection and intervention [13]. Several studies have explored the potential of BrAce measurements as a non-invasive tool for detecting DKA in patients with type 1 and type 2 diabetes, particularly those using SGLT2 inhibitors. Sha et al. (2022) [13] demonstrated that their breath analysis device effectively tracked ketone body production in individuals undergoing fasting, managing diabetes, or receiving SGLT2 inhibitor therapy. Similarly, Edelman et al. (2019) [41] found a significant correlation between BrAce levels and blood ketone concentrations in type 2 diabetes patients treated with SGLT2 inhibitors, underscoring its potential for ketosis monitoring. Further supporting this, Saasa et al. (2019) [42] reported an association between BrAce levels and ketosis, highlighting the clinical value of BrAce as a biomarker for assessing DKA risk, particularly in diabetic patients using SGLT2 inhibitors. The availability of non-invasive, real-time, and portable BrAce devices also empowers patients to actively manage their health, fostering greater engagement, compliance, and improved health outcomes [37,38]. Although further comprehensive clinical studies are required to fully validate the utility of breath acetone measurement in individuals using SGLT2 inhibitors, early evidence indicates its potential as a valuable tool for the early detection of ketosis in this population [38].

## 5. Conclusions

This systematic review highlights the potential of nanotechnology-based breath analysis as a viable, non-invasive method for ketone monitoring in patients with T1DM. The strong correlation observed between BrAce levels and blood ketone levels supports BrAce as an accurate proxy for blood-based assessments, presenting a promising alternative to the invasive capillary tests currently used for detecting and managing DKA. Advances in sensor technology have significantly enhanced sensitivity, with studies reporting detection rates as high as 94.7%. This capability not only facilitates early intervention but also reduces the risk of severe DKA episodes and associated hospitalizations, delivering both clinical and economic benefits. Despite these promising findings, certain limitations must be addressed to unlock the full potential of BrAce technology in clinical practice. Variability in methodologies, definitions, and BrAce cut-off values across studies poses challenges for device comparability, underscoring the need for standardisation in future research. Establishing uniform measurement protocols, sensitivity thresholds, and cut-off values will improve device reliability, streamline regulatory approval processes, and facilitate broader clinical adoption.

Current devices also face performance challenges under extreme conditions, such as severe DKA, where high acetone levels or other interfering compounds may impact sensor accuracy. Large-scale studies are necessary to refine sensor algorithms and enhance selectivity for acetone amidst other VOCs. Personalising devices by accounting for individual variability and environmental factors could further improve their precision. As nanotechnology-based breath analysis advances, its applications should extend beyond T1DM to address other ketotic states, including those triggered by ketogenic diets, fasting, and medications like SGLT2 inhibitors. Exploring these broader applications could enhance its utility and reinforce its role in diverse clinical and metabolic scenarios.

In conclusion, nanotechnology-based BrAce measurement represents a promising advancement in non-invasive ketone monitoring, with the potential to revolutionise DKA management. Addressing current limitations and achieving standardisation in methodologies, sensitivity thresholds, and device calibration will be essential for the development of reliable, user-friendly tools. Such advancements could empower individuals with T1DM to monitor ketone levels more effectively, enabling timely intervention and reducing the risk of severe DKA episodes. The progression toward rapid, real-time breath analysis offers significant promise for enhancing clinical outcomes and improving the quality of care for those at risk of DKA.

## Figures and Tables

**Figure A1:**
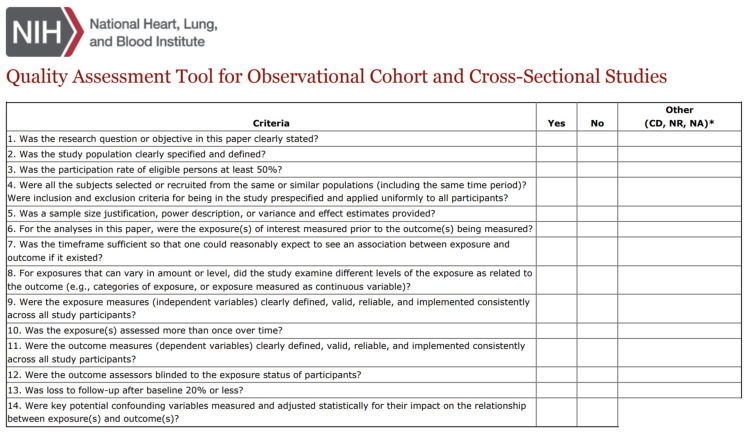
National Heart, Lung, and Blood Institute (NHLBI) Quality Assessment Tool for Observational Cohort and Cross-Sectional Studies. * CD, cannot determine; NA, not applicable; NR, not reported.

## Data Availability

The original contributions presented in the study are included in the article. Further inquiries can be directed to the corresponding author, Kamal Marfatia.

## Abbreviations

T1DM	Type I diabetes mellitus
T2DM	Type 2 diabetes mellitus
DKA	Diabetic ketoacidosis
SGLT2i	Sodium glucose transport protein 2 inhibitors
ED	Emergency department
ICU	Intensive care unit
TKB	Total ketone body in blood
PPM	Parts per million
BrAce	Breath acetone
PICO	Population, Intervention, Comparative Interventions, and Outcomes

## Appendix A

**Table A1:** Search terms for Medline OVID and Embase.

Search Numbers	Terms
1	Diabetes Mellitus, Type 1.mp. [mp = title, book title, abstract, original title, name of substance word, subject heading word, floating sub-heading word, keyword heading word, organism supplementary concept word, protocol supplementary concept word, rare disease supplementary concept word, unique identifier, synonyms, population supplementary concept word, anatomy supplementary concept word]
2	ketosis/or diabetic ketoacidosis/or diabetes mellitus/or hyperglycaemia/
3	Breath Tests/
4	ketones/or acetone/or ketone bodies/or 3-hydroxybutyric acid/or acetoacetates/
5	diagnosis/or emergency treatment/or glycaemic control/or hospitalisation
6	1 and 2 and 4
7	2 and 3 and 4
8	1 and 2 and 3
9	2 and 4 and 5
10	6 and 7
11	3 and 4

**Table A2:** Search terms for Cochrane.

Search Numbers	Terms
1	(juvenile diabetes):ti,ab,kw OR (Diabetes Mellitus type 1) ti,ab,kw OR (DMT1):ti,ab,kw
2	(ketones) ti,ab,kw OR (acetone) ti,ab,kw OR (ketone bodies) ti,ab,kw OR (3-hydroxybutyric acid) ti,ab,kw OR (acetoacetates) ti,ab,kw
3	(1 and 2)
4	MeSH descriptor: [Breath Tests] explode all trees
5	(breath):ti,ab,kw OR (exhaled):ti,ab,kw OR (exhalation):ti,ab,kw OR (expired gas):ti,ab,kw OR (expired air):ti,ab,kw OR (nanotechnology) ti,ab,kw
6	4 OR 5
7	(diagnosis) ti,ab,kw OR (emergency treatment) ti,ab,kw OR (glycaemic control) ti,ab,kw OR (hospitalisation) ti,ab,kw
8	2 OR 7
9	3 and 6 and 8

**Table A3:** PICO tool.

PICO Tool	Search Terms
**Patient population:** Diabetes mellitus type 1	Juvenile diabetes, IDDM1,DMT1, type 1 diabetes mellitus
AND	
**Intervention/Exposure:** BrAce nanosensors	Breath nanosensors, breath analysis, breath biomarkers, VOC, breathalyser, Non-invasive ketone measurement, Ketone detection, nanosensor technology, exhaled air, exhalation, expired gas, expired air
AND	
**Comparison:** Blood/plasma ketone levels	3HB, 3-hydroxybutyric, acetoacetates, ketones, ketone bodies
AND	
**Outcome measure:** Ketosis/DKA	Ketosis, diabetic ketoacidosis, hyperglycaemia, DKA, ketones, ketone bodies, acetone, breath acetone, BrAce, Breath

## Appendix B

**Table A4:** Risk of Bias Assessment.

Cohort studies—NHLBI risk of bias tool															Total/14	Overall study quality
**Author (year)**	Q1	Q2	Q3	Q4	Q5	Q6	Q7	Q8	Q9	Q10	Q11	Q12	Q13	Q14		
**Hancock 2020 [28]**	🗸	🗸	🗸	🗸	🗶	🗸	🗸	🗸	🗸	🗸	🗸	🗸	NA	🗸	12	Good
**Suntrup 2020 [34]**	🗸	🗸	🗸	🗶	🗸	🗶	🗸	🗶	🗸	🗸	🗶	🗶	NA	🗸	8	Fair
**Blaikie 2014 [29]**	🗸	🗸	🗸	🗸	🗸	🗶	🗶	🗶	🗸	🗶	🗸	🗶	NA	🗸	8	Fair
Cross-Sectional studies—NHLBI risk of bias tool															Total/14	Overall study quality
**Author (year)**	Q1	Q2	Q3	Q4	Q5	Q6	Q7	Q8	Q9	Q10	Q11	Q12	Q13	Q14		
**Sha 2022 [13]**	🗸	🗶	🗸	🗸	🗸	🗶	🗸	🗶	🗸	🗸	🗸	🗶	NA	🗸	9	Fair
**Tsunemi 2022 [31]**	🗸	🗸	🗸	🗸	🗸	🗶	🗸	🗶	🗸	🗸	🗸	🗶	NA	🗸	10	Good
**Gunter 2022 [32]**	🗸	🗸	🗸	🗸	🗸	🗶	🗸	🗸	🗸	🗸	🗸	🗶	NA	🗸	11	Good
All other types—NHLBI risk of bias tool															Total/14	Overall study quality
**Author (year)**	Q1	Q2	Q3	Q4	Q5	Q6	Q7	Q8	Q9	Q10	Q11	Q12	Q13	Q14		
**Ruhani 2017 [33]**	🗸	🗶	NA	🗶	🗶	🗶	🗸	🗸	🗶	🗸	🗶	🗶	NA	🗸	5	Poor
**Wang 2008 [14]**	🗸	🗸	🗸	🗶	🗶	🗸	🗸	🗸	🗸	🗸	🗸	NA	NA	🗸	10	Good
**Fan 2014 [35]**	🗶	🗶	🗶	🗶	🗶	🗶	🗸	🗸	🗸	🗶	🗸	🗶	NA	🗸	5	Poor
**Reyes-Reyes 2014 [7]**	🗸	🗸	🗸	🗸	🗶	🗸	🗶	🗶	🗸	🗸	🗸	🗶	NA	🗸	9	Fair
**Akturk 2021 [30]**	🗸	🗸	🗶	🗸	🗸	🗶	🗸	🗶	🗶	🗸	🗶	🗶	🗶	🗶	6	Fair
